# Modelling of ultrasonic assisted osmotic dehydration of cape gooseberry using adaptive neuro-fuzzy inference system (ANFIS)

**DOI:** 10.1016/j.ultsonch.2023.106425

**Published:** 2023-04-29

**Authors:** Kshirod Kumar Dash, Anjelina Sundarsingh, G.V.S. BhagyaRaj, Vinay Kumar Pandey, Béla Kovács, Shaikh Ayaz Mukarram

**Affiliations:** a Department of Food Processing Technology, Ghani Khan Choudhury Institute of Engineering and Technology (GKCIET), Malda, West Bengal 732141, India; b Department of Bioengineering, Integral University, Lucknow, Uttar Pradesh, India; c Faculty of Agriculture, Food Science and Environmental Management Institute of Food Science, University of Debrecen, Debrecen 4032, Hungary

**Keywords:** Cape gooseberry, Ultrasonication, Osmotic dehydration, ANFIS

## Abstract

In the present investigation, the cape gooseberry (*Physalis peruviana* L.) was preserved by the application of osmotic dehydration (sugar solution) with ultrasonication. The experiments were planned based on central composite circumscribed design with four independent variables and four dependent variables, which yielded 30 experimental runs. The four independent variables used were ultrasonication power ($X_{P}$) with a range of 100–500 W, immersion time ($X_{T}$) in the range of 30–55 min, solvent concentration ($X_{C}$) of 45–65 % and solid to solvent ratio ($X_{S}$) with range 1:6–1:14 w/w. The effect of these process parameters on the responses weight loss ($Y_{W}$), solid gain ($Y_{S}$), change in color ($Y_{C}$) and water activity ($Y_{A}$) of ultrasound assisted osmotic dehydration (UOD) cape gooseberry was studied by using response surface methodology (RSM) and adaptive neuro-fuzzy inference system (ANFIS). The second order polynomial equation successfully modeled the data with an average coefficient of determination ($R^{2}$) was found to be 0.964 for RSM. While for the ANFIS modeling, Gaussian type membership function (MF) and linear type MF was used for the input and output, respectively. The ANFIS model formed after 500 epochs and trained by hybrid model was found to have average $R^{2}$ value of 0.998. On comparing the $R^{2}$ value the ANFIS model found to be superior over RSM in predicting the responses of the UOD cape gooseberry process. So, the ANFIS was integrated with a genetic algorithm (GA) for optimization with the aim of maximum $Y_{W}$ and minimum $Y_{S}$, $Y_{C}$ and $Y_{A}$. Depending on the higher fitness value of 3.4, the integrated ANFIS-GA picked the ideal combination of independent variables and was found to be $X_{P}$ of 282.434 W, $X_{T}$ of 50.280 min, $X_{C}$ of 55.836 % and $X_{S}$ of 9.250 w/w. The predicted and experimental values of response at optimum condition predicted by integrated ANN-GA were in close agreement, which was evident by the relative deviation less than 7%.

## Highlights

• In Ultrasonic assisted osmotic dehydration (UOD) high-frequency sound waves cause cavitation.
• Ultrasonication at power 280 W was effective for higher water loss in cape gooseberry.
• UOD inactivates microorganisms and enzymes in cape gooseberry, enhancing self-life.
• ANFIS can model complex UOD involving nonlinear and uncertain relationships.

## Graphical abstract

**Figure:**
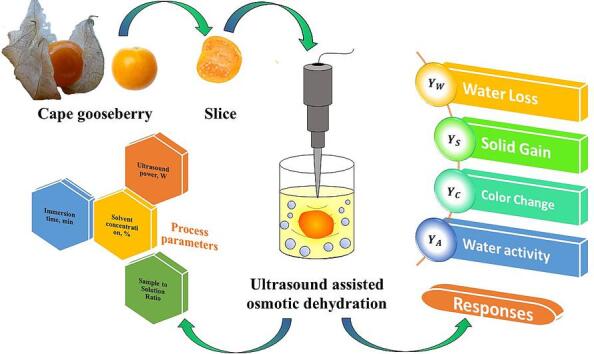

## 1 Introduction

Cape gooseberry (*Physalis peruviana* L.) is a subtropical quick growing herbaceous, semi-shrub, upright perennial plant in the *Solanaceae* family and genus *Physalis* [36]. Cape gooseberry is a climacteric fruit grown in many countries over the world and is known by different names Poha berry, Peruvian Ground berry, Uvilla, and Rashbhari [13]. Cape gooseberry is an exotic fruit that contains minerals, vitamins C, and antioxidant compounds such as anthocyanins, carotenoids, tannins, alkaloids, and flavonoids [14]. The bioactive compounds in the berries are reported to offer some health benefits and have been widely used in traditional medicine to treat a variety of diseases [29], [30]. Cape gooseberry is very appealing to customers due to its therapeutic activities, such as antioxidant, antimicrobial, antipyretic, anti-inflammatory, anti-allergic, and anti-ulcer [3], [12]. Besides these health benefits, the berries were underutilized due to the perishable nature as it has higher water activity which permits deterioration quickly [22]. So, the berry requires proper preservation methods without degrading the nutritional and quality factors.

Several dehydration methods can be employed to preserve fruits and vegetables to extend their shelf life. Traditional treatments such as hot air ovens and solar drying have been used since time immemorial [21]. However, since fruits contain antioxidants, vitamins, and phenolic acids that are significantly affected by thermal treatment, alternatives to these are an emerging trend in preserving fruit and vegetable [43]. One such technique that is preferred for reducing the water activity of fruits and vegetables is osmotic dehydration, a non-thermal treatment [2]. Several studies have found it to be beneficial by many researches in reducing the moisture of different fruits such as persimmon fruit [11]; apples [44]; black jamun fruit [45]. Osmotic dehydration was favored to preserve the structure, flavor, and aroma of agricultural produce because it only uses an osmotic medium to remove water from fruits [37]. The process includes a counter-current mass transfer in which the solute from the osmotic solution moves inside the fruit while moisture or water is drawn out. The osmotic medium is typically a hypertonic solution that aids in fruit shrinkage by removing moisture, which is possible due to the difference in water chemical potential between the solution and the product [32].

In compared to other dehydration methods, OD is generally a very slow-moving process that needs to be accelerated by combining other techniques such as hot air [16], vacuum[26], ultrasound[19], high hydrostatic pressure[27], pulsed electric field[17].

Among the discussed techniques, ultrasound application and osmotic dehydration were the most dependable and non-thermal methods. This procedure employs the use of ultrasound, which uses sound waves to generate energy at inaudible frequencies in human ears [31], [37]. Ultrasound aided osmotic dehydration (UOD) has been shown to expedite the mass transfer and improve dehydrated product quality [32]. Cavitation is created when ultrasound is applied, which can be beneficial when transferring water. The creation of channels caused by cell disruption after cavitation increases effective water diffusivity. When ultrasound is passed through a liquid medium, it causes mechanical agitation, resulting in acoustic cavitation leading to the physical structure of the fruit. The micro channels formed by mechanical agitation facilitate the passage of water molecules from within the product to the fruit's surface, increasing water diffusivity. The recent applications of ultrasound assisted osmotic dehydration for improving the shelf life of perishable fruits such as dragon fruit [9]; plum [38]; kiwi [42]; white mulberry [39], cranberries [33]. Both ultrasound and osmotic dehydration increase the amount of solid and liquid transmission between the hypertonic solution and the sample [4], [11].

The different independent variables of ultrasound assisted osmotic dehydration, such as ultrasonication power, ultrasound frequency, temperature, osmotic agent, hypertonic solution concentration, solid to liquid ratio, and sample thickness affect the quality of the samples. The effect of these process parameters on the dehydrated sample can be studied by applying different modeling techniques such as mathematical models, response surface methodology (RSM), artificial neural network (ANN), and adaptive neuro-fuzzy interface (ANFIS). Out of all these techniques, ANFIS is the more versatile and effective modeling tool that can be applied effectively for complex non-linear problems like drying, osmotic dehydration, and extraction of phytochemicals from agricultural produce to develop a relationship between input and responses [48].

Based on this, the present study aims to investigate the effect of ultrasound on the osmotic dehydration of cape gooseberry and modeling using RSM and ANFIS. Both the models were compared with each other, and the best model was coupled with genetic algorithm for optimizing the ultrasound assisted osmotic dehydration technique. The process parameters considered for the study were ultrasonication power, time, solution concentration, and solid to solvent ratio and were modeled to know their effect on water loss, solid gain, color change, and water activity of UOD cape gooseberry.

## 2 Material and method

### 2.1 Raw materials

Fresh cape gooseberries (*Physalis peruviana L*.) were purchased from Malda, West Bengal, and kept at 4 °C until the experiments. The cape gooseberries were washed, peeled, and cut into cube-shaped slices of dimension 10 mm.

### 2.2 Osmotic dehydration

The hypertonic solution was prepared by mixing sugar with distilled water, the solution was transferred to a beaker, and the cube-shaped slice of cape gooseberry was dipped into the solution. The solution containing the sample was treated with ultrasound in an ultrasonicator equipped with the probe at room temperature. After each osmotic dehydration treatment, the samples were quickly and gently removed from the sugar solution, washed with distilled water, blotted with absorbent paper to remove any excess osmotic solution that had adhered to the surface, and weighed. For a better outcome, the experiments were carried out in duplicate. The samples were analyzed for weight loss ($Y_{W}$), solid gain ($Y_{S}$), change in color ($Y_{C}$) and water activity ($Y_{A}$). The experiments were performed based on CCD design.

### 2.3 Modeling of ultrasound assisted osmotic dehydration

#### 2.3.1 Experimental design

The experimental study was investigated by applying the design of experiments approach employing central composite circumscribed design (CCD). Two different modeling approaches, response surface methodology (RSM), and adaptive neuro-fuzzy inference system (ANFIS), were implemented to model the UOD of the cape gooseberry process to investigate the effect of each independent variable on the dependent variable. The experimental design consists of four independent variables with five levels and four dependent variables. The independent variables selected were ultrasonication power ($X_{P}$), immersion time ($X_{T}$), solvent concentration ($X_{C}$), and solid to solvent ratio ($X_{S}$) with a range of these parameters are 100–500 W, 30–55 min, 45–65 %, and 1:6–1:14 w/w, respectively, presented in Table 1. The range of each variable was chosen based on preliminary experiments performed, and a total of 30 experimental runs were performed according to the design. The best model was used to optimize the process by integrating it with the genetic algorithm on comparing the statistical parameters.

**Table 1:** Independent variables range in coded and real values along with their notation and units for ultrasound assisted osmotic dehydration of cape gooseberry.

Independent variable	Unit	Range and Levels				
		−2	−1	0	1	2
Ultrasonic Power ($X_{P}$)	W	100	200	300	400	500
Time ($X_{T}$)	min	35	40	45	50	55
Sugar concentration ($X_{C}$)	%	45	50	55	60	65
Solid to solution ratio ($X_{R}$)	w/w	1:06	1:08	1:10	1:12	1:14

#### 2.3.2 Response surface methodology

For the RSM modelling, a generalised second-degree polynomial equation, as given in Eq. (1), was fitted to each response to study the effect of variables and to mathematically describe the process[6].

(1) $$Y=\beta_{o}+\sum_{i=1}^{n}\beta_{i}x_{i}+\sum_{i=1}^{n-1}\sum_{j=i+1}^{n}\beta_{\mathrm{ij}}x_{i}x_{j}+\sum_{i=1}^{n}\beta_{\mathrm{ii}}x_{i}^{2}$$

Where $\beta_{o}$*,* $\beta_{i}$*,* $\beta_{\mathrm{ii}}$ and $\beta_{\mathrm{ij}}$ are constant coefficient for coded independent variables, and n is the number of independent variables i.e., 4. The equation presented in Eq. (1) can be represented as presented in Eq. (2), which is a more contextual polynomial equation fitted for the osmotic dehydration condition for cape gooseberries.

(2) $$Y=\beta_{0}+\beta_{1}x_{P}+\beta_{2}x_{T}+\beta_{3}x_{C}+\beta_{4}x_{S}+\beta_{12}x_{P}x_{T}+{\beta_{13}x}_{P}x_{C}+\beta_{14}x_{P}x_{S}+\beta_{23}x_{T}x_{C}+{\beta_{23}x}_{T}x_{C}+\beta_{34}x_{C}x_{S}+\beta_{11}{x_{P}}^{2}+\beta_{22}{x_{T}}^{2}+\beta_{33}{x_{C}}^{2}+\beta_{44}{x_{S}}^{2}$$

Where Y is the function of the independent variables such as ultrasonic power, immersion time, concentration, and sample to solvent ratio.

#### 2.3.3 ANFIS modelling for osmotic dehydration of cape gooseberry

**Fig 1:**
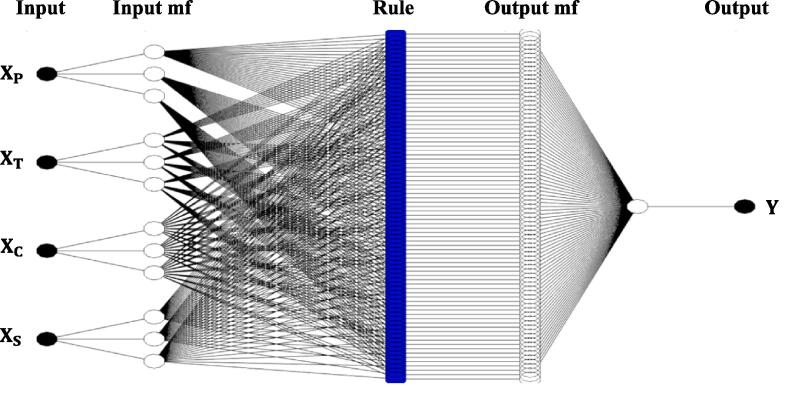
Proposed ANFIS architecture for ultrasound assisted osmotic dehydration of cape gooseberry.

The osmotic dehydration of cape gooseberry with the application of ultrasound waves was modeled by ANFIS based on the method followed by Taghinezhad et al. [47]. ANFIS architecture with five layers was presented in Fig. 1, and was achieved using Fuzzy Logic Toolbox by Matlab software (MATLAB 2018). The CCD design yielded experimental runs that were divided into 70% used for training with the help of the First order Takagi-Sugeno model and hybrid algorithm. The remaining 30% of the data was assigned for testing of the formed ANFIS. Four ANFIS models were developed for each response by considering one response at a time in the output layer (fifth layer). Each architecture consists of four process parameters as inputs (first layer). In the second layer, each process parameter was assigned three membership function nodes, and each node was connected to a set of rules in the third layer, resulting in 81 rules. The membership function (MFs) used in the second and fourth layers of input and output were chosen by the values of the coefficient of determination ($R^{2}$) and root mean square error (RMSE) during training. The effect of independent variables on the responses was estimated by following the method described by Raj & Dash [40].

### 2.4 Determination of responses

#### 2.4.1 Water loss ($Y_{W}$) and solid gain ($Y_{S}$)

Water loss and solid gain of cape gooseberry were calculated by using the equations presented in Eq. (3), (4), respectively [24]

(3) $$\mathrm{WL}\left(\%\right)=\frac{M_{0}w_{0}-M_{t}w_{t}}{M_{0}}\times 100$$

(4) $$SG\left(\%\right)=\frac{M_{t}s_{t}-M_{0}s_{0}}{M_{0}}\times 100$$

Where $M_{0}$ indicates the initial weight of cape gooseberry, g; $M_{t}$ indicates the weight of cape gooseberry after each osmotic treatment, g; $w_{0}$ and $w_{t}$ indicates the initial and final water content of cape gooseberry, respectively, g/g; $s_{0}$ and $s_{t}$ indicates the initial and final dry matter content of cape gooseberry, respectively, g/g wet basis. The dry matter and water content of the cape gooseberry sample were calculated using AOAC method.

#### 2.4.2 Color change ($Y_{C}$)

The color of cape gooseberry was analyzed using a Hunter lab colorimeter (Ultrascan VIS, Hunter Lab., Inc., and Reston, VA). The L* (lightness), a* (redness and greenness) and b* (yellowness and blueness) values for each sample were recorded in replicates for both fresh and dried fruit. The color change ($Y_{C}$) was determined with the help of equation Eq. (5) [15].

(5) $$Y_{C}=\sqrt{\left(L-L_{0}\right)+\left(a-a_{0}\right)+\left(b-b_{0}\right)}$$

Where, $L_{0}$_,_ $a_{0}$ , $b_{0}$ are the color values for fresh Cape gooseberries.

#### 2.4.3 Water activity ($Y_{A}$)

Water activity ($Y_{A}$) of UOD sample is determined as the ratio of the vapor pressure of UOD treated cape goose berry to the vapor pressure of pure water. The water activity of UOD cape gooseberry was measured using a water activity meter (Aqua lab Dew Point 4TE, USA), and the measurements were conducted in triplicates.

### 2.5 Multi-objective genetic algorithm

The best model output was combined with a multi-objective genetic algorithm (GA) to optimize the ultrasound-assisted osmotic dehydration of the cape gooseberry. GA works on the principle of ‘survival of the fittest’ strategy by means of selection, reproduction, crossover, and mutation of the initial population[40]. The fitness function (FF) was used to optimize the UOD process by maximizing the responses water loss and minimizing the responses solid gain, change in color and water activity designated in Eq. (6).

(6) $$FF=\left\{\begin{array}{c} \max Y_{W}\left(X_{P},X_{T},X_{C},X_{R}\right)\\ \min Y_{S}\left(X_{P},X_{T},X_{C},X_{R}\right)\\ \min Y_{C}\left(X_{P},X_{T},X_{C},X_{R}\right)\\ \min Y_{A}\left(X_{P},X_{T},X_{C},X_{R}\right)\\ 100W\leq X_{P}\leq 500W\\ 35min\leq X_{T}\leq 55min\\ 45\%\leq X_{C}\leq 65\%\\ 1:06\leq X_{S}\leq 1:14 \end{array}\right)$$

### 2.6 Statistical tools

The statistical parameters calculated in the present investigation were the coefficient of determination ($R^{2}$), root mean square error (RMSE) and relative deviation (Rd) for validating the adequacy of the models used and were presented in Eq. (7), Eq. (8), and Eq. (9), respectively[8].

(7) $$R^{2}=1-\frac{\sum_{i=1}^{n}\left(Y_{i}-Y_{j}\right)}{\sum_{i=1}^{n}\left(Y_{k}-Y_{e}\right)}$$

(8) $$RMSE=\sqrt{\frac{\sum_{i=1}^{n}\left(Y_{i}-Y_{j}\right)}{n-1}}$$

(9) $$R_{d}=\frac{100}{n-1}\sum_{i=1}^{n}\frac{\left|{Y_{i}-Y}_{j}\right|}{Y_{j}}\;$$

where $Y_{i}$ indicates values obtained from the model, $Y_{j}$ indicates the values of the experiment, $Y_{k}$ indicates the mean value, and n is the number of observations.

## 3 Results and discussion

### 3.1 Modeling of UOD process

#### 3.1.1 Modeling by RSM

The experimental data of the response water loss was fitted to the second order by non-linear regression analysis. The F-value for the model and lack of fit (error) was found to be 67.426 and 4.516, respectively. According to the significance level, the model p-value was less than 0.05 and the lack of fit was higher than 0.05, indicating that the model was significant and the lack of fit was insignificant at p = 0.05. The $R^{2}$ was found to be 0.976, which signifies that the second order polynomial equation represented in Eq. (2) was found to predict the data with higher accuracy. The adj-$R^{2}$ value alters the $R^{2}$ value based on the sample size and the number of terms in the model. The value of adj $R^{2}$ was 0.960, also high to advocate for a high significance the model and the error RMSE value was found to be 0.096. The coefficient of variation (CV%) indicates the relative dispersion of the experimental points from the predictions of the second-order polynomial models. CV is also low as 2.05. This implies that the differences between experimental and expected values are small. Adequate precision is measured by the signal-to-noise ratio, which should be greater than 4. The ratio in this work is determined to be 30.582, indicating a sufficient signal.

**Fig. 2:**
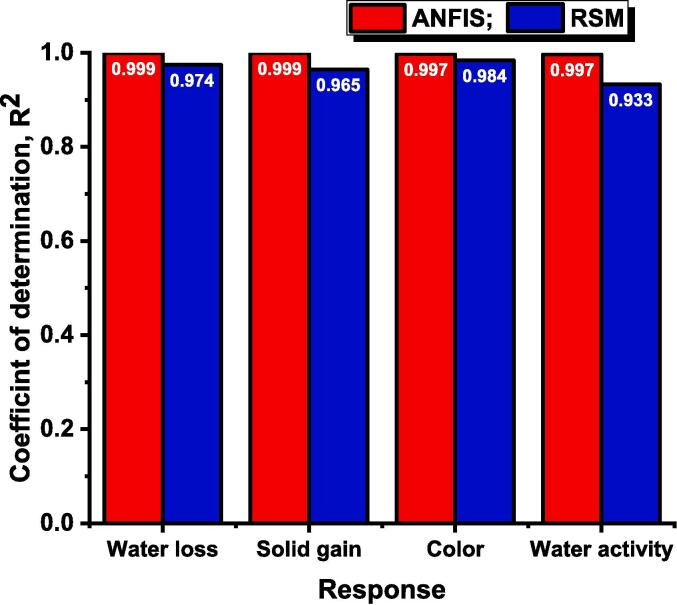
Coefficient of determination of RSM and ANFIS model designed for ultrasound assisted osmotic dehydration of cape gooseberry.

**Fig. 3:**
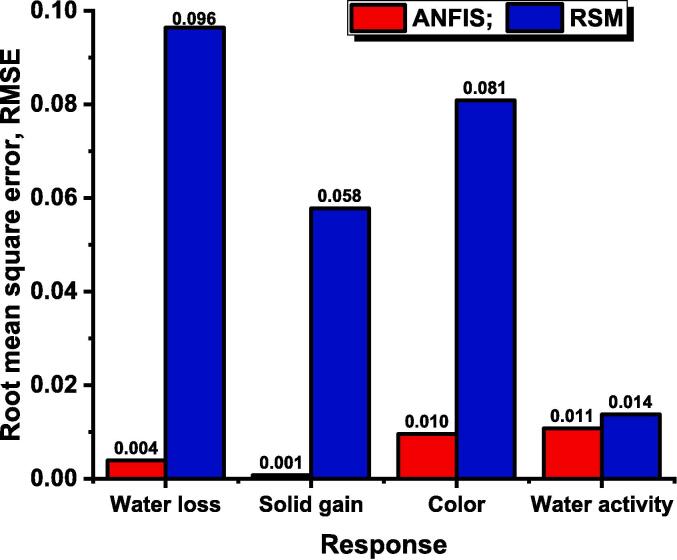
Root mean square error of RSM and ANFIS model designed for ultrasound assisted osmotic dehydration of cape gooseberry.

Similarly, for the other three responses, the model was significant (p<0.05), and the lack of fit was insignificant (p>0.05) which was desirable for the validation of the polynomial equation. The $R^{2}$ value for the response solid gain, change in color, and water activity was found to be 0.965, 0.984, and 0.933, respectively, illustrated in Fig. 2. The RMSE value for the same sequence was observed to be 0.058, 0.081 and 0.014 respectively shown in Fig. 3. The adj $R^{2}$ was higher than 0.870 and close to the respective response $R^{2}$. The CV% was lower than 7.058, and adequate precision was higher than 13.604. The statistical parameters of the RSM model indicate that the second order polynomial equation was found to model the experimental data with higher accuracy and lower error. Various statistical parameters and the coefficients of the polynomial equation of the four responses presented in Eq. (2), were presented in Table 2.

**Table 2:** The analysis of variance for fitting the second order polynomial models to experimental data for Water loss, solid gain, and color change and water activity.

Source	Coefficient Estimate			
	Water loss ($Y_{W}$)	Solid gain ($Y_{S}$)	Change in color ($Y_{C}$)	Water activity ($Y_{A}$)
Intercept				
$\beta_{0}$	33.337	6.329	14.604	0.972
$x_{P}$($\beta_{1}$)	$7.605^{*}$	$3.598^{*}$	$8.482^{*}$	$-0.013^{*}$
$x_{T}$($\beta_{2}$)	$2.244^{*}$	$1.313^{*}$	$1.399^{*}$	$-0.003^{***}$
$x_{C}$($\beta_{3}$)	$2.804^{*}$	$4.041^{*}$	$1.954^{*}$	$-0.011^{*}$
$x_{S}$($\beta_{4}$)	$1.483^{*}$	$0.366^{ns}$	$-0.159^{ns}$	$-0.004^{**}$
Interaction terms				
$x_{P}x_{T}$($\beta_{12}$)	$1.764^{***}$	$3.003^{*}$	$4.782^{*}$	$-0.002^{\mathrm{ns}}$
$x_{P}x_{C}$($\beta_{13}$)	$2.443^{**}$	$2.557^{*}$	$5.133^{*}$	$-0.006^{**}$
$x_{P}x_{S}$($\beta_{14}$)	$0.509^{ns}$	$0.243^{ns}$	$1.008^{ns}$	$0.001^{\mathrm{ns}}$
$x_{T}x_{C}$($\beta_{23}$)	$-1.082^{ns}$	$-0.868^{ns}$	$-2.058^{*}$	$0.002^{\mathrm{ns}}$
$x_{T}x_{S}$($\beta_{24}$)	$-0.170^{ns}$	$0.088^{ns}$	$-0.303^{ns}$	$0.001^{\mathrm{ns}}$
$x_{C}x_{S}$($\beta_{34}$)	$-1.401^{ns}$	$-0.298^{ns}$	$-0.192^{ns}$	$0.001^{\mathrm{ns}}$
Quadratic terms				
$x_{P}^{2}$($\beta_{11}$)	$1.829^{**}$	$0.806^{ns}$	$4.396^{*}$	$-0.001^{\mathrm{ns}}$
$x_{T}^{2}$($\beta_{22}$)	$-0.281^{ns}$	$0.856^{ns}$	$3.456^{*}$	$0.001^{\mathrm{ns}}$
$x_{C}^{2}$($\beta_{33}$)	$2.585^{*}$	$1.456^{**}$	$4.782^{*}$	$-0.003^{***}$
$x_{S}^{2}$($\beta_{44}$)	$0.759^{ns}$	$3.456^{*}$	$4.006^{*}$	$-0.004^{***}$
Model (F value)	$67.426^{*}$	$55.333^{*}$	$176.655^{*}$	$14.867^{*}$
Lack of fit (F value)	$4.516^{\mathrm{ns}}$	$4.709^{\mathrm{ns}}$	$1.767^{\mathrm{ns}}$	$1.793^{\mathrm{ns}}$
$R^{2}$	0.974	0.965	0.984	0.933
Adj.$R^{2}$	0.960	0.953	0.978	0.870
Predicted.$R^{2}$	0.907	0.888	0.961	0.676
C.V. %	2.049	7.058	2.761	0.689
Adeq. Precision	30.582	26.924	48.454	13.604
Std. Dev.	2.049	7.058	2.761	0.007

#### 3.1.2 Modeling by ANFIS

The best ANFIS model for ultrasound assisted osmotic dehydration of cape gooseberry was formed with Gaussian type and linear type membership functions for input MF and output MF respectively. After 500 epochs, the training data for the best ANFIS model was found to have $R^{2}$ value close to unity ($R^{2}>0.99$) and RMSE values lower than $0.14\times 10^{-2}$ that indicates the prediction capability of the model. The $R^{2}$ and RMSE value of testing data for ANFIS model formed for the responses weight loss ($Y_{W}$), solid gain ($Y_{S}$), change in color ($Y_{C}$) and water activity ($Y_{A}$) was found to have 0.998 and 0.015, 0.997 and 0.006, 0.991 and 0.032, and 0.985 and 0.039, respectively. The overall $R^{2}$ (Fig. 2) and RMSE (Fig. 3) value (training and testing) of the ANFIS model for the responses $Y_{W}$, $Y_{S}$, $Y_{C}$ and $Y_{A}$ was observed to be 0.999 and 0.004, 0.999 and 0.001, 0.997 and 0.010, and 0.997 and 0.011, respectively. The statistical parameters of the ANFIS ($R^{2}>0.997$) revealed the superiority over the RSM model ($R^{2}>0.933$) in predicting the responses at different combinations of independent variables within the selected range. Comparable results of higher predicting capability of drying characteristics were reported for the ANFIS model with $R^{2}$ close to unity and RMSE less than 0.072 during convective hot air drying of potato, garlic, and cantaloupe [23]. Therefore, the formed four ANFIS models were further applied to determine the relative influence of process parameters on response and the optimization of the process parameters according to Eq. (6).

### 3.2 Modeling for water loss

**Fig. 4:**
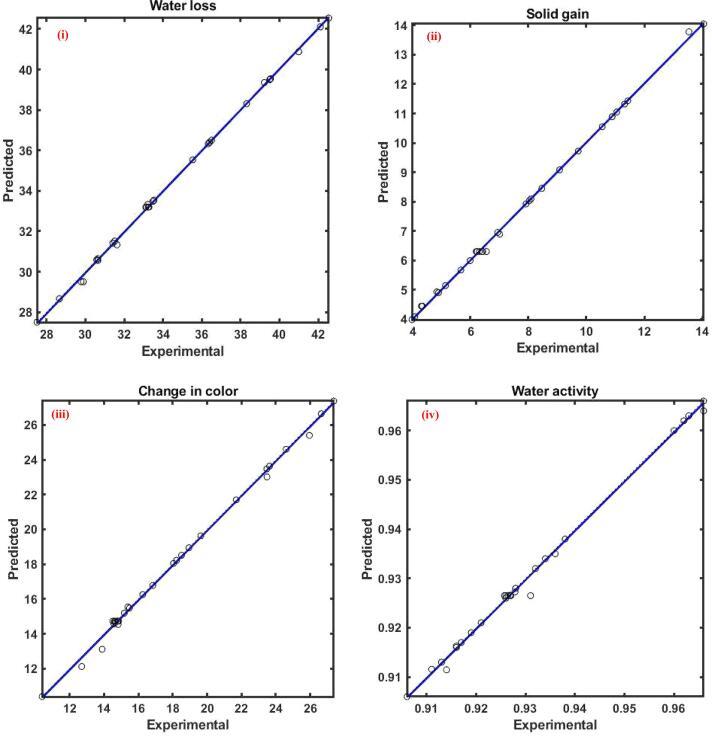
Experimental Vs ANFIS model predicted values for (i) water loss; (ii) solid gain; (iii) change in color; and (iv) water activity of ultrasound assisted osmotic dehydration of cape gooseberry.

Water loss signifies the removed water from the plant tissues due to the pressure difference between the sample and osmotic solution and is a vital characteristic during the process. The water loss from the cape gooseberry during OD by the application of ultrasound at various combinations of independent variables was observed to be in the range of 27.5–42.54. The plot between observed water loss data and ANFIS predicted water loss data is shown in Fig. 4(i). The correlation coefficient was close to one, indicating that both data were in close agreement with each other. Table 3 shows the effects of independent variables on water loss of cape gooseberry during the osmotic dehydration process using the ANFIS model.

**Table 3:** Relative influence of the process parameters on the response of ultrasound assisted osmotic dehydration of cape gooseberry.

Parameter	$x_{P}$	$x_{T}$	$x_{C}$	$x_{S}$
$y_{W}$	1.846	0.774	0.822	0.266
$y_{S}$	1.353	0.614	1.292	0.165
$y_{C}$	2.000	0.398	0.579	−0.106
$y_{A}$	−1.667	−0.733	−1.214	−0.867

According to Table 3, ultrasonication power has the greatest positive effect on cape gooseberry weight loss, with the value of 1.846. The other three parameters, sugar concentration, time, and sample to solid ratio, were also observed to positively influence the water loss with the values 0.774, 0.822, and 0.266, respectively. Higher value signifies the greater influence on the water loss. From the relative effect, it was also evident that all four process parameters positively influenced the water loss. So, increasing any process parameter will result in increased water loss from the cape gooseberry. The increased ultrasonic power can raise the temperature of the solution, causing more improved drive of free water from the tissues of cape gooseberry triggered by the bulging of the cell membrane and plasticizing effect that might be the reason for improved water loss. The other reason may be due to the acoustic cavitation formed during ultrasonic treatment had a sponge-like effect on the surface of the sample, causing alternate compressive and expansion stress. This resulted in the complete collapse of the sample’s semi-permeable membrane, resulting in the development of microscopic channels within the sample through which water could escape [35]. The increase in temperature of the solvent because of ultrasound when combined with a higher concentration of the hypertonic solution and longer dehydration time during osmotic dehydration can attribute to accelerated dehydration. Osmotic dehydration with ultrasound as a pretreatment process carried out on pumpkin seeds also reported a similar trend where both treatment time and concentration of osmotic solution have a positive influence on water loss [7].

Similar results of improved water loss were reported with increasing the immersion time to the hypertonic solution during OD of kiwi fruits[10]. This might be due to the weakening of the semipermeable membrane of the cape gooseberry. A similar trend was observed in research conducted on pulsed vacuum osmotic dehydration (PVOD) to investigate its effect on cape gooseberry at a temperature of 40 °C by Zapata et al. [52], where a positive influence of osmotic agent concentration on the water loss of the cape gooseberry was reported up to 70°Brix, but further increase in concentration has a negative impact on the water loss. This trend may be due to the interaction of the two factors, namely concentration and temperature, and the fact that their increases favor the exit of water from the fruit. Another study by Rahman et al. (2019) found that increasing the concentration of the glucose and sugar solution in plums increased water loss through the application of ultrasound during osmotic dehydration. Comparable trends were observed where the increase of sample to osmotic solution from 1:5 to 1:13 improved the loss of water from the amla cubes during OD using sucrose as osmotic agent [49].

### 3.3 Modeling for solid gain

The maximum and minimum solid gain for the UOD cape gooseberry sample was found to be 14.05 and 3.987, respectively. The effects of independent variables on the solid gain of cape gooseberry during osmotic dehydration by ultrasound process according to the ANFIS model were shown in Table 3. The regression plot between the experimental and ANFIs predicted solid gain at different blends of process parameters was illustrated in Fig. 4(ii). The relative magnitude of the independent variables indicates that the factors showing the maximum effect on solid gain during the UOD process in the order as: ultrasonic power (1.353), followed by sugar concentration (1.292), treatment time (0.614) and solid to solvent ratio (0.165). The selected process parameters were observed to positively influence the solid gain, which was noticeable by the positive symbol of the relative magnitude. The positive effect indicates that an increase of any process parameter enhances the solid gain in the UOD sample.

The influence of ultrasound during osmotic dehydration on solid gain was in agreement with the results reported by Li et al. [25], where the increase in ultrasonication power from 800 to 1600 W increased the solid gain during UOD of Chinese yam. The increase in ultrasonication power might have formed the microscopic channels in the sample, which enhanced the mass transfer between the sample and the hypertonic solvent [51]. The positive effect of ultrasonication power on the water loss and solid gain was in agreement with the results reported for the ultrasound-assisted osmotic dehydration of pakchoi stems [50].

Zapata et al. [52] found that the concentration of the osmotic solution has the lowest negative effect on solid gain, followed by temperature and pressure. This indicates that a concentrated osmotic solution will result in the formation of a membrane barrier that will not facilitate the passage of solids. While this might have happened due to the combination of parameters like temperature and pressure, in this case, we have observed the highest positive effect of $X_{C}$ with a magnitude of 2.19 for solid gain. The difference in results obtained in the two cases might be due to the different factors and processes being used for optimization.

The positive influence of treatment time on the solid gain was in accordance with the research work reported by Prithani & Dash [37], where a significant increase of 74.28% in solid gain in the initial phase was followed by a 14% increase in the second phase for ultrasound assisted osmotic dehydration of Kiwi fruit. Similar solid gain and water loss trends were observed with increasing sample immersion time to the hypertonic solution during OD of banana[41].

### 3.4 Modeling for color change

The physical parameter color is one of the vital quality attributes for the acceptability of dehydrated agricultural produce. Compared with the thermally dried product, the osmotically dehydrated product can produce high quality product with lower damage to the color parameters preventing enzymatic browning[34]. In the present investigation, the higher change in color with reference to the fresh sample was observed to be 27.37, whereas the lower value of change in color was approximately 62% lesser than the highest change in color. A lower value of change in color indicates higher quality and vice versa. The values of change in color at various combinations of independent variables were plotted against the predicted values of the ANFIS model and were shown in Fig. 4(iii). Table 3 depicts the effects of independent variables on color change of cape gooseberry during ultrasound assisted osmotic dehydration process. According to the relative influence, ultrasonication power (2.00) was found to have a higher impact on the response $Y_{C}$ which means the increase of ultrasonication power contributed to the higher change in color with reference to the fresh sample. The relative effect value of other parameters time, solvent concentration and solid to solvent ratio was observed to be 0.398, 0.579, and −0.106, respectively. The negative symbol of solid to solvent ratio indicates that the increase of solid to solvent ratio decreased the response change in color. Ultrasound had a positive effect on the change in color, which might be due to the rise in temperature of the solvent with the increase of the parameter. Color changes in green fruits and vegetables are normally associated with the degradation of compounds like chlorophyll, which are often affected by temperature [28].

Li et al. [25] have reported an increase in L* and a* values with an increase in ultrasonication power during ultrasound assisted osmotic dehydration of Chinese yam, which might be explained by the fact that a reduction in moisture changes the optical properties of a food sample. Solvent concentration and time of dehydration have been shown to exert a positive effect on the color change of the cape gooseberry. The drying of blueberries using osmosis as a pre-treatment has shown similar results, where an increase of change in color from 1.20 to 1.05 was reported in with an increase of time of treatment from 3 to 12 h, respectively [46]. The result indicated the change in color was indeed a factor of time which can further be described by the combination of concentration of osmotic solution and time with ultrasonication power resulting in a higher change in color. Similar results have been reported where ultrasound assisted osmotic dehydration significantly increased all three color parameters (L*, a* & b*) of persimmon fruit [11]. The positive influence of process variable $X_{S}$ might be due to the Millard reaction, where the higher concentrations of sugars can cause this reaction more easily because of the thick coating of osmotic agent on the surface of the sample [1].

### 3.5 Modeling for water activity

The water activity ($Y_{A}$) of a product is a measure of the availability of water for degradation reactions. In general, the process of osmotic dehydration of agricultural produce should decrease the water activity of the product. The water activity for the UOD treated cape gooseberry sample was observed to be in the range of 0.904 to 0.964. The experimental water activity was plotted against corresponding ANFIS predicted water activity, presented in Fig. 4(iv). The effects of independent variables on the water activity of cape gooseberry during the osmotic dehydration process according to the ANFIS model were depicted in Table 3.

While it can be clearly seen from Table 3 that all the process variables had a negative relationship with $Y_{A}$, which was evident by the negative symbol for the calculated relative effect value. This trend of process variables on the water activity was desirable during osmotic dehydration, where all the process parameters should contribute to lowering the response with increase in any process parameter. Out of all the four process parameters, ultrasonication power had a higher effect with a relative value of 1.667, followed by sugar concentration (1.214), solid to solvent ratio (0.867), and immersion time (0.733). Other than the process parameters, the loss of water or the gain of solid is also reported to have negative influence on the water activity.

With the increase in ultrasonication power, the temperature increase should have facilitated moisture loss and solid gain, resulting in lower water activity. The effect of process parameters on the water activity was in accordance with the results reported during the osmotic dehydration of potatoes [18]. The phenomena can further be explained by the unique characteristics of sugar solutions that facilitate easy diffusion, resulting in reduced water activity, which happens due to the driving force for dehydration.

Atarés et al. [5] also reported a decrease in water activity with increasing the immersion time of apple cylinders of 10 mm diameter and height in glucose solution (osmotic solution) during osmotic dehydration. The rise in the independent variable $X_{S}$ in the solution may be accelerated the driving force to remove water from the sample, which resulted in the decrease in water activity [18], [20].

### 3.6 Optimisation of osmotic dehydration for cape gooseberry (*P. peruviana*)

The output of the ANFIS model was fed to the genetic algorithm as the initial population, and the optimal conditions for osmotic dehydration of cape gooseberry were determined to achieve maximum value of water loss and minimum value of solid gain, change in color, and water activity, according to the fitness function indicated in equation Eq. (6). The best solution was selected based on the fitness values of 3.4 and the optimum condition for the process variables ultrasonication power ($X_{P}$), time ($X_{T}$), solvent concentration ($X_{C}$), and solid to solvent ratio ($X_{S}$) was observed to be 282.434 W, 50.280 min, 55.836 %, and 9.250 w/w, respectively. The integrated ANFIS-GA model predicted the responses weight loss ($Y_{W}$), solid gain ($Y_{S}$), change in color ($Y_{C}$) and water activity ($Y_{A}$) for the UOD of cape goose berry was 41.351, 11.071, 13.088, and 0.910, respectively, shown in Table 4. The observed values of responses at the best condition were found to be $Y_{W}$ of 42.746 ± 1.455, $Y_{S}$ of 10.525 ± 0.215, $Y_{C}$ of 13.942 ± 0.540 and $Y_{A}$ of 0.926 ± 0.049 and were validated by comparing with the predicted values. The relative deviation between the observed and ANFIS-GA forecasted values of $Y_{W}$, $Y_{S}$, $Y_{S}$ and $Y_{A}$ was found to be 3.263%, 5.190%, 6.125%, and 1.773%, respectively, presented in Table 4. Based on the percentage of Rd, both the data obtained at the optimum level were in good agreement with each other.

**Table 4:** Experimental and ANFIS-GA predicted values at optimum condition.

Response	Experimental	Predicted	Rd, %
Water loss ($Y_{W}$)	42.746 ± 1.455	41.351	3.263
Solid gain ($Y_{S}$)	10.525 ± 0.215	11.071	5.190
Change in color ($Y_{C}$)	13.942 ± 0.540	13.088	6.125
Water activity ($Y_{A}$)	0.926 ± 0.049	0.910	1.773

## 4 Conclusion

The modeling of the UOD cape gooseberry was carried out by RSM and ANFIS to study the effect of process parameters such as ultrasonication power, treatment time, osmotic solution concentration, and sample to solution ratio on the responses water loss, solid gain, color change, and water activity. Based on the statistical parameter, the ANFIS was found to predict the responses with higher accuracy and lower error when compared with RSM. When the ultrasonic power and osmotic solution concentration were increased, there was a significant increase in water loss and solid gain. Further, the ANFIS was integrated with GA for optimization of independent processing parameters of ultrasound assisted osmotic dehydration of cape gooseberry. The optimization was performed with the goal of maximizing water loss while minimizing solid gain, change in color, and water activity. The best solution was selected based on the fitness values of 3.4 and the optimum condition for the process variables ultrasonication power ($X_{P}$), time ($X_{T}$), solvent concentration ($X_{C}$), and solid to solvent ratio ($X_{S}$) was observed to be 282.434 W, 50.280 min, 55.836 %, and 9.250 w/w, respectively. The relative deviation between the observed and predicted data at optimum levels of process parameters was less than 7% which signifies a close agreement between them. The findings aided in determining the effect of ultrasound-assisted osmotic dehydration on water loss and solid gain.

## Funding

Project No. TKP2021-NKTA-32 has been implemented with support from the National Research, Development and Innovation Fund of Hungary, financed under the TKP2021-NKTA funding scheme.

## Declaration of Competing Interest

The authors declare that they have no known competing financial interests or personal relationships that could have appeared to influence the work reported in this paper.

## References

[1] Abrahão F.R., Corrêa J.L.G. (2021). Osmotic dehydration: More than water loss and solid gain. Crit. Rev. Food Sci. Nutr..

[2] Ahmed I., Qazi I.M., Jamal S. (2016). Developments in osmotic dehydration technique for the preservation of fruits and vegetables. Innov. Food Sci. Emerg. Technol..

[3] Alp S., Ercisli S., Dogan H., Temim E., Leto A., Zia-Ul-Haq M., Hadziabulic A., Aladag H. (2016). Chemical composition and antioxidant activity Ziziphora clinopodioides ecotypes from Turkey. Romanian Biotechnological Letters.

[4] Ashokkumar M. (2015). Applications of ultrasound in food and bioprocessing. Ultrason. Sonochem..

[5] Atarés L., Chiralt A., González-Martínez C. (2008). Effect of solute on osmotic dehydration and rehydration of vacuum impregnated apple cylinders (cv. Granny Smith). J. Food Eng..

[6] Bardhan P., Baruah J., Raj G.V.S.B., Kalita E., Mandal M. (2021). Optimization of culture conditions for biomass and lipid production by oleaginous fungus Penicillium citrinum PKB20 using response surface methodology (RSM). Biocatal. Agric. Biotechnol..

[7] Bchir B., Bouaziz M.A., Ettaib R., Sebii H., Danthine S., Blecker C., Besbes S., Attia H. (2020). Optimization of ultrasound-assisted osmotic dehydration of pomegranate seeds (Punica granatum L.) using response surface methodology. J. Food Process. Preserv..

[8] Bhagya Raj G.V.S., Dash K.K. (2021). Heat transfer analysis of convective and microwave drying of dragon fruit. J. Food Process Eng.

[9] Bhagya Raj G.V.S., Dash K.K. (2022). Ultrasound assisted osmotic dehydration of dragon fruit slices: Modeling and optimization using integrated artificial neural networks and genetic algorithms. J. Food Process. Preserv..

[10] Bialik M., Wiktor A., Latocha P., Gondek E. (2018). Mass transfer in osmotic dehydration of kiwiberry: Experimental and mathematical modelling studies. Molecules.

[11] Bozkir H., Rayman Ergün A., Serdar E., Metin G., Baysal T. (2019). Influence of ultrasound and osmotic dehydration pretreatments on drying and quality properties of persimmon fruit. Ultrason. Sonochem..

[12] Bravo K., Osorio E. (2016). Characterization of polyphenol oxidase from Cape gooseberry (Physalis peruviana L.) fruit. Food Chem..

[13] Chandra J., Prasad V.M., Bahadur V., Mishra A. (2021). Study on response of different doses of nitrogen on vegetative growth, flowering, fruiting and fruit quality of Cape gooseberry (Physalis peruviana L.). Pharma Innov. J.

[14] Darwish M.S., Qiu L., Taher M.A., Zaki A.A., Abou-Zeid N.A., Dawood D.H., Shalabi O.M.A.K., Khojah E., Elawady A.A. (2022). Health Benefits of Postbiotics Produced by E. coli Nissle 1917 in Functional Yogurt Enriched with Cape Gooseberry (Physalis peruviana L.). Fermentation.

[15] Dash K.K., Shangpliang H., Bhagya Raj G.V.S., Chakraborty S., Sahu J.K. (2021). Influence of microwave vacuum drying process parameters on phytochemical properties of sohiong (Prunus nepalensis) fruit. J. Food Process. Preserv..

[16] Dehghannya J., Hosseinlar S.H., Heshmati M.K. (2018). Multi-stage continuous and intermittent microwave drying of quince fruit coupled with osmotic dehydration and low temperature hot air drying. Innov. Food Sci. Emerg. Technol..

[17] Dermesonlouoglou E., Chalkia A., Dimopoulos G., Taoukis P. (2018). Combined effect of pulsed electric field and osmotic dehydration pre-treatments on mass transfer and quality of air dried goji berry. Innov. Food Sci. Emerg. Technol..

[18] Eren I., Kaymak-Ertekin F. (2007). Optimization of osmotic dehydration of potato using response surface methodology. J. Food Eng..

[19] Fernandes F.A.N., Braga T.R., Silva E.O., Rodrigues S. (2019). Use of ultrasound for dehydration of mangoes (Mangifera indica L.): kinetic modeling of ultrasound-assisted osmotic dehydration and convective air-drying. J. Food Sci. Technol..

[20] Hamdan M., Sharif A.O., Derwish G., Al-Aibi S., Altaee A. (2015). Draw solutions for Forward Osmosis process: Osmotic pressure of binary and ternary aqueous solutions of magnesium chloride, sodium chloride, sucrose and maltose. J. Food Eng..

[21] Hasan M.U., Malik A.U., Ali S., Imtiaz A., Munir A., Amjad W., Anwar R. (2019). Modern drying techniques in fruits and vegetables to overcome postharvest losses: A review. J Food Process Preserv.

[22] Junqueira J.R.d.J., Corrêa J.L.G., de Oliveira H.M., Ivo Soares Avelar R., Salles Pio L.A. (2017). Convective drying of cape gooseberry fruits: Effect of pretreatments on kinetics and quality parameters. LWT Food Sci. Technol..

[23] Kaveh M., Rasooli Sharabiani V., Amiri Chayjan R., Taghinezhad E., Abbaspour-Gilandeh Y., Golpour I. (2018). ANFIS and ANNs model for prediction of moisture diffusivity and specific energy consumption potato, garlic and cantaloupe drying under convective hot air dryer. Information Processing in Agriculture.

[24] Li L., Yu Y., Xu Y., Wu J., Yu Y., Peng J., An K., Zou B., Yang W. (2021). Effect of ultrasound-assisted osmotic dehydration pretreatment on the drying characteristics and quality properties of Sanhua plum (Prunus salicina L.). Lwt.

[25] Li L., Zhang M., Wang W. (2020). Ultrasound-assisted osmotic dehydration pretreatment before pulsed fluidized bed microwave freeze-drying (PFBMFD) of Chinese yam. Food Biosci..

[26] Liu Z.L., Staniszewska I., Zielinska D., Zhou Y.H., Nowak K.W., Xiao H.W., Pan Z., Zielinska M. (2020). Combined Hot Air and Microwave-Vacuum Drying of Cranberries: Effects of Pretreatments and Pulsed Vacuum Osmotic Dehydration on Drying Kinetics and Physicochemical Properties. Food Bioproc. Tech..

[27] Luo W., Tappi S., Wang C., Yu Y., Zhu S., Dalla Rosa M., Rocculi P. (2019). Effect of High Hydrostatic Pressure (HHP) on the Antioxidant and Volatile Properties of Candied Wumei Fruit (Prunus mume) During Osmotic Dehydration. Food Bioproc. Tech..

[28] Manolopoulou E., Varzakas T.h., Petsalaki A. (2016). Chlorophyll Determination in Green Pepper Using two Different Extraction Methods. Current Res. Nutrition Food Sci. J..

[29] Marchioretto L.D.R., De Rossi A., Conte E.D. (2020). Chemical root pruning improves quality and nutrient uptake of Cape Gooseberry (Physalis peruviana) seedlings. Sci. Hortic..

[30] Muniz J., Kretzschmar A.A., Rufato L., Pelizza T.R., Rufato A.D.R., de Macedo T.A. (2014). General aspects of physalis cultivation. Ciência Rural.

[31] Noshad M., Mohebbi M., Shahidi F., Mortazavi S.A. (2012). Effect of osmosis and ultrasound pretreatment on the moisture adsorption isotherms of quince. Food Bioprod. Process..

[32] Nowacka, M., Dadan, M., & Tylewicz, U. (2021). Current applications of ultrasound in fruit and vegetables osmotic dehydration processes. In *Applied Sciences (Switzerland)* (Vol. 11, Issue 3, pp. 1–22). https://doi.org/10.3390/app11031269.

[33] Nowacka M., Fijalkowska A., Dadan M., Rybak K., Wiktor A., Witrowa-Rajchert D. (2018). Effect of ultrasound treatment during osmotic dehydration on bioactive compounds of cranberries. Ultrasonics.

[34] Nowacka M., Tylewicz U., Romani S., Dalla Rosa M., Witrowa-Rajchert D. (2017). Influence of ultrasound-assisted osmotic dehydration on the main quality parameters of kiwifruit. Innov. Food Sci. Emerg. Technol..

[35] Oladejo A.O., Ma H. (2016). Optimisation of ultrasound-assisted osmotic dehydration of sweet potato (Ipomea batatas) using response surface methodology. J. Sci. Food Agric..

[36] Olivares-Tenorio M.-L., Dekker M., Verkerk R., van Boekel M.A.J.S. (2016). Health-promoting compounds in cape gooseberry (Physalis peruviana L.): Review from a supply chain perspective. Trends Food Sci. Technol..

[37] Prithani R., Dash K.K. (2020). Mass transfer modelling in ultrasound assisted osmotic dehydration of kiwi fruit. Innov. Food Sci. Emerg. Technol..

[38] Rahaman A., Zeng X.-A., Kumari A., Rafiq M., Siddeeg A., Manzoor M.F., Baloch Z., Ahmed Z. (2019). Influence of ultrasound-assisted osmotic dehydration on texture, bioactive compounds and metabolites analysis of plum. Ultrason. Sonochem..

[39] Rahimi N., Ahraritabas A., Ansarifar E. (2022). Optimization of Ultrasound – Assisted Osmotic Dehydration of White Mulberry. J. Food Process. Preserv..

[40] Raj G.V.S.B., Dash K.K. (2022). Microencapsulation of Dragon Fruit Peel Extract by Freeze-Drying Using Hydrocolloids: Optimization by Hybrid Artificial Neural Network and Genetic Algorithm. Food Bioproc. Tech..

[41] Rascón M.P., Huerta-Vera K., Pascual-Pineda L.A., Contreras-Oliva A., Flores-Andrade E., Castillo-Morales M., Bonilla E., González-Morales I. (2018). Osmotic dehydration assisted impregnation of Lactobacillus rhamnosus in banana and effect of water activity on the storage stability of probiotic in the freeze-dried product. Lwt.

[42] Roueita G., Hojjati M., Noshad M. (2020). Study of Physicochemical Properties of Dried Kiwifruits Using the Natural Hypertonic Solution in Ultrasound-assisted Osmotic Dehydration as Pretreatment. Int. J Fruit Sci..

[43] Sakooei-Vayghan R., Peighambardoust S.H., Hesari J., Peressini D. (2020).

[44] Samborska K., Eliasson L., Marzec A., Kowalska J., Piotrowski D., Lenart A., Kowalska H. (2019). The effect of adding berry fruit juice concentrates and by-product extract to sugar solution on osmotic dehydration and sensory properties of apples. J. Food Sci. Technol..

[45] Sharma M., Dash K.K. (2019). Effect of ultrasonic vacuum pretreatment on mass transfer kinetics during osmotic dehydration of black jamun fruit. Ultrason. Sonochem..

[46] Stojanovic J., Silva J.L. (2007). Influence of osmotic concentration, continuous high frequency ultrasound and dehydration on antioxidants, colour and chemical properties of rabbiteye blueberries. Food Chem..

[47] Taghinezhad E., Kaveh M., Szumny A. (2021). Optimization and prediction of the drying and quality of turnip slices by convective-infrared dryer under various pretreatments by rsm and anfis methods. Foods.

[48] Tao Y., Li Y., Zhou R., Chu D.T., Su L., Han Y., Zhou J. (2016). Neuro-fuzzy modeling to predict physicochemical and microbiological parameters of partially dried cherry tomato during storage: effects on water activity, temperature and storage time. J. Food Sci. Technol..

[49] Tiroutchelvame D., Sivakumar V., Maran P. (2015). Mass transfer kinetics during osmotic dehydration of amla (Emblica officinalis L.) cubes in sugar solution. Chem. Ind. Chem. Eng. Q..

[50] Wu X.F., Zhang M., Mujumdar A.S., Yang C.H. (2020). Effect of ultrasound-assisted osmotic dehydration pretreatment on the infrared drying of Pakchoi Stems. Drying Technol..

[51] Xin Y., Zhang M., Adhikari B. (2013). Effect of trehalose and ultrasound-assisted osmotic dehydration on the state of water and glass transition temperature of broccoli (Brassica oleracea L. var. botrytis L.). J. Food Eng..

[52] Zapata M.J.E., Ciro G.G.L., Marulanda L.P. (2016). Optimización de la deshidratación osmótica a vacío pulsante de uchuva (Physalis peruviana L.) por medio de la metodología de superficies de respuesta. Agronomia Colombiana.

